# Supplementary material for: The Evolution and Characterization of the RNA Interference Pathways in Lophotrochozoa
Source: Genome Biol Evol. 2024 May 7;16(5):evae098. doi: 10.1093/gbe/evae098 (PMC11114477; doi:10.1093/gbe/evae098)
Supplement: evae098_Supplementary_Data [file evae098_supplementary_data.zip › Supplementary_materials.zip!/selected_miRNAs/NoGe_selected_miRNAs/mirdeep_pdf_files/NMRB01002279.1_Notospermus_geniculatus_strain_Ushimado_scaffold2279,_whole_genome_shotgun_sequence_37128.pdf]

Provisional ID	:	NMRB01002279.1_Notospermus_geniculatus_strain_Ushimado_scaffold2279,_whole_genome_shotgun_sequence_37128
Score total	:	538.9
Score for star read(s)	:	3.9
Score for read counts	:	530.3
Score for mfe	:	1.6
Score for randfold	:	0
Score for cons. seed	:	3
Total read count	:	1052
Mature read count	:	828
Loop read count	:	0
Star read count	:	224

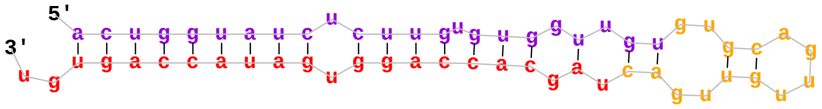

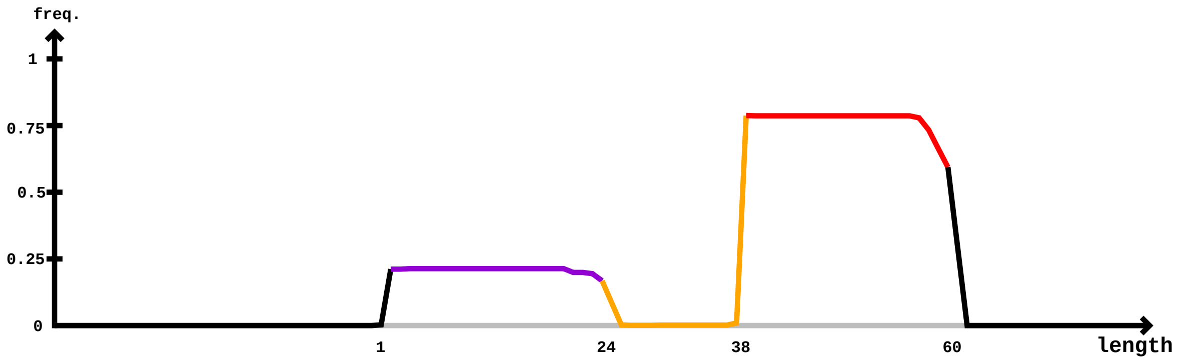


```
                                   Star                                Mature
5'- gauacugugcuuuaagcuuccuccuccucacucacugguaucucuugugugguugugugcaguuguugacuagcaccaggugauaccagugugcgaguugguucaugcuaug   -3'   obs
    gauacugugcuuuaagcuuccuccuccucacucacugguaucucuugugugguugugugcaguuguugacuagcaccaggugauaccagugugcgaguugguucaugcuaug         exp
    ..............(((.....((..(((.(.((((((((((.((((.(((.(.((..((....))..)).).))))))).))))))))))).).)))..)).....)))...    reads   mm   sample
    .................................acugguaucucuugugGgguug.........................................................    1       1    Mm1
    .................................acugguaucucuuguguUguug.........................................................    1       1    Mm1
    .................................acugguaucucuugugugguug.........................................................    13      0    Mm1
    .................................acugguaucucuuguguCguugu........................................................    1       1    Mm1
    .................................acugguaucucuugugugguugu........................................................    43      0    Mm1
    .................................acugguaucucuugugAgguugu........................................................    1       1    Mm1
    ......................................................................uagcaccaggugauaccagA......................    8       1    Mm1
    ......................................................................uagcaccaggugauaccagu......................    11      0    Mm1
    ......................................................................uagcaccaggugauaccagug.....................    9       0    Mm1
    ......................................................................uagcaccaggugauaccagugu....................    56      0    Mm1
    ......................................................................uagcaccaggugauacAagugu....................    1       1    Mm1
    ......................................................................uagcGccaggugauaccagugu....................    1       1    Mm1
    ......................................................................uagcaccaggGgauaccagugu....................    1       1    Mm1

    ................................cacugguaucucuugugugguu..........................................................    3       0    Mm2
    .................................acugguaucucuugugugguug.........................................................    2       0    Mm2
    .................................acugguaucucuugugugguugu........................................................    54      0    Mm2
    .................................acugguaucucuuguguUguugu........................................................    1       1    Mm2
    .................................Ncugguaucucuugugugguugu........................................................    1       1    Mm2
    .................................acugguaucuAuugugugguugu........................................................    1       1    Mm2
    .................................acugguaucucuugugugguuguU.......................................................    1       1    Mm2
    .................................acugguaucucuugugugguugugugcaguuguugacu.........................................    1       0    Mm2
    ...................................ugguaucucuugugugguugugu......................................................    2       0    Mm2
    ......................................................................uagcaccaggugauaccG........................    1       1    Mm2
    ......................................................................uagcaccaggugauacca........................    1       0    Mm2
    ......................................................................uagcaccaggugauaccag.......................    1       0    Mm2
    ......................................................................uagcaccaggugauaccagu......................    3       0    Mm2
    ......................................................................uagcaccaggugauaccaUu......................    1       1    Mm2
    ......................................................................uagcaccaggugauacAagu......................    1       1    Mm2
    ......................................................................uagcaccaggugauaccagug.....................    10      0    Mm2
    ......................................................................uagcaccaggugauacUagug.....................    1       1    Mm2
    ......................................................................uagcaccaggugauacUagugu....................    3       1    Mm2
    ......................................................................uagcaccaggugauaccUgugu....................    1       1    Mm2
```

Star Mature

```
gauacugugcuuuaagcuuccuccuccucacucacugguaucucuugugugguugugugcaguuguugacuagcaccaggugauaccagugugcgaguugguucaugcuaug
......................................................................Nagcaccaggugauaccagugu....................  2    1  Mm2
......................................................................uagcaccaggugauaccagugu....................  98   0  Mm2
......................................................................uagcaccaggugauaccCgugu....................  1    1  Mm2
......................................................................uagcaccaggugauaccagugG....................  10   1  Mm2
......................................................................uagcaccaggugauaccaguguA...................  3    1  Mm2
......................................................................uagcaccaggugauaccaguguU...................  4    1  Mm2

.....................................................................cuagcaccaggugauaccaguU.....................  2    1  Fm1
......................................................................uagcaccaggugauacca........................  5    0  Fm1
......................................................................uagcaccaggugauaccaU.......................  1    1  Fm1
......................................................................uagcaccaggugauaccagu......................  11   0  Fm1
......................................................................uagcaccaggugauaccagug.....................  5    0  Fm1
......................................................................uagcaccaggugauacUagugu....................  1    1  Fm1
......................................................................uagcaccaggugauGccagugu....................  1    1  Fm1
......................................................................uagcaccaggugauaccagugu....................  174  0  Fm1
......................................................................uagUaccaggugauaccagugu....................  1    1  Fm1
......................................................................uagcaccaggugauaccCgugu....................  1    1  Fm1
......................................................................uagcaccaggugauaccagugG....................  15   1  Fm1
......................................................................uagcaccaggugauaccGgugu....................  1    1  Fm1
......................................................................uagcaccaggugauaccaguguU...................  10   1  Fm1
......................................................................uagcaccaggugauaccaguguA...................  7    1  Fm1

.................................acugguaucucuugugugguugu........................................................  14   0  Fm2
.....................................................................cuagcacAaggugauaccagugu....................  1    1  Fm2
.....................................................................cuagcaccaggugauaccagugu....................  4    0  Fm2
.....................................................................cuagcaccaggugauaccagugG....................  1    1  Fm2
......................................................................uagcaccaggugauaccaguU.....................  4    1  Fm2
......................................................................uagcaccaggugauaccagug.....................  1    0  Fm2
......................................................................uagcaccaggugauaccagugG....................  3    1  Fm2
......................................................................uagcaccaggugauaccagugu....................  52   0  Fm2
......................................................................Cagcaccaggugauaccagugu....................  1    1  Fm2
......................................................................uagcaccGggugauaccagugu....................  1    1  Fm2
......................................................................uagcaccaggugauaccaguguU...................  13   1  Fm2

.................................acugguaucucuugugugg............................................................  1    0  Mm3
.................................acugguaucucuuguguggC...........................................................  1    1  Mm3
.................................acugguaucucuugugugguu..........................................................  1    0  Mm3
.................................acugguaucucuugugugguugu........................................................  39   0  Mm3
.................................acugguaucucuugugugguAgu........................................................  1    1  Mm3
.................................acugguaucucuugugugguuguU.......................................................  2    1  Mm3
.............................................................guuguugacuagcaccaggugauaccagugu....................  1    0  Mm3
......................................................................uagcaccaggugauacca........................  1    0  Mm3
......................................................................uagcaccaggugauaccagu......................  8    0  Mm3
......................................................................uagcaccaggugauacGagu......................  1    1  Mm3
......................................................................uagcaccaggugauaccagGg.....................  1    1  Mm3
......................................................................uagcaccaggugauaccagug.....................  1    0  Mm3
......................................................................uagcaccaggugauaccagugG....................  2    1  Mm3
......................................................................uagcaccaggugauaccaguUu....................  1    1  Mm3
......................................................................uagcaccaggugauaccagugu....................  26   0  Mm3
......................................................................uagcaccaggugauaccagugC....................  1    1  Mm3
......................................................................uagcaccaggugauaccaguAu....................  1    1  Mm3
......................................................................uagcaccaggugauaccaguguU...................  1    1  Mm3

.................................acugguaucucuugugugg............................................................  14   0  Fm3
.................................acugguaucucuugugugguug.........................................................  11   0  Fm3
.................................acugguaucuAuugugugguugu........................................................  1    1  Fm3
.................................acugguaucucuugugugguugu........................................................  13   0  Fm3
.................................acugguaucucuugugugguuguU.......................................................  1    1  Fm3
......................................................................uagcaccaggugauacUag.......................  1    1  Fm3
......................................................................uagcaccaggugauacGag.......................  1    1  Fm3
......................................................................uagcaccaggugauaccag.......................  44   0  Fm3
......................................................................uagcaccaggugauaccagu......................  28   0  Fm3
......................................................................uagcaccagguUauaccagu......................  1    1  Fm3
......................................................................uagcaccaggugauacUagu......................  1    1  Fm3
......................................................................uagcaccaggugauaccagug.....................  39   0  Fm3
......................................................................uagcaccaggugauaccagugu....................  119  0  Fm3
......................................................................uagcaccaggugauacUagugu....................  1    1  Fm3
......................................................................uagcaccaggugauaccUgugu....................  1    1  Fm3
......................................................................uagcaccaggugauaccaguguU...................  4    1  Fm3
```
